# Supplementary material for: Effect of habitual reading direction on saccadic eye movements: A pilot study
Source: PLoS One. 2023 Jun 2;18(6):e0286801. doi: 10.1371/journal.pone.0286801 (PMC10237664; doi:10.1371/journal.pone.0286801)
Supplement: S1 Appendix — (DOCX) [file pone.0286801.s001.docx]

**Appendix I: Comparison of results between 2 eye trackers (Eyelink 1000 vs. Eyelink Portable Duo)**

Mixed analysis of variance (ANOVA) with 2 eye trackers (Eyelink 1000 vs. Eyelink Portable Duo) and group (LTR vs. RTL) as between-subject factors on prosaccade latency (S1 Fig 1.), prosaccade gain (S1 Fig 2.) and antisaccade latency (S1 Fig 3.) was conducted.

**S1 Fig 1. Effect of different trackers on prosaccade latency**





*Average prosaccade latency with standard deviations was plotted for the LTR (i.e. Chinese participants) and RTL groups (i.e. Arabic and Persian participants) using 2 different eye trackers (Eyelink 1000 vs. Eyelink Portable Duo). Statistically, no significant main effect of trackers was found on prosaccade latency (F(1, 28)=0.64, p=0.43). The interaction between the trackers and group was not significant (F(1, 28)=0.07, p=0.80). Prosaccade latency recorded by the Eyelink 1000 and Eyelink Portable Duo was similar (178.84 ± 17.11 vs. 185.23 ± 29.05 msec).*

**S1 Fig 2. Effect of different trackers on prosaccade gain**





*Similar results were found on prosaccade gain. No significant main effect of eye trackers (F(1, 28)=0.43, p=0.52) or interaction effect (F(1, 28)=0.09, p=0.77) was found. Prosaccade gain was similar between the Eyelink 1000 and Eyelink Portable Duo (0.99 ± 0.07 vs. 0.97 ± 0.13).*

**S1 Fig 3. Effect of different trackers on antisaccade latency**





*Neither trackers (F(1, 28)=2.01, p=0.17) nor interaction (F(1, 28)=0.00, p=0.99) had significant effects on antisaccade latency towards correct directions. The latency was similar between the 2 eye trackers (272.93 ± 28.06 vs. 292.63 ± 48.74 msec).*
