# Supplementary material for: Effect of habitual reading direction on saccadic eye movements: A pilot study
Source: PLoS One. 2023 Jun 2;18(6):e0286801. doi: 10.1371/journal.pone.0286801 (PMC10237664; doi:10.1371/journal.pone.0286801)
Supplement: Attachment — Submitted filename: Response to reviewer.docx [file pone.0286801.s002.docx]

**Response to reviewers**

Manuscript PONE-D-22-00817

Dear Dr. Maiello and reviewer,

Thank you for giving us the opportunity to submit the revised draft of the manuscript entitled “Effect of habitual reading direction on saccadic eye movements: A pilot study” for publication as a registered report in PLoS One. We appreciate your time and effort to provide feedback on our manuscript. The comments are valuable, improving the quality of our paper. We have made the corresponding changes in our manuscript, which are highlighted in yellow. Please also see below for the point-to-point responses to reviewer’s comments and concerns.

**Reviewer #1’ s comments:**

***While I have no doubts that this was a well-designed and carefully conducted experiment, I do have a couple of reservations about the manuscript that I would like to see addressed before being able to make a recommendation for publication. My main reservations are power and transparency throughout the manuscript. I will describe these points below. I would like to disclose that I typically investigate eye movements during reading and, as such, my comments are seen through that lens.***

Thank you for reviewer’s very positive comment!

***1. My main concern with this study is power. The authors note that 16 participants per between-subjects factor will provide 85% power at a one-tailed alpha of 0.05 with an effect size of 0.71. This is fair, but what unit is 0.71? Is the partial eta or Cohen’s d? Also, why is this random value chosen? It’s not justified at all. This makes me wonder if it is posthoc power that is far from optimal and arguable is a fallacy. Furthermore, the authors use multiple analyses to address single predictions. Therefore, the alpha value should be adjusted for multiple comparisons. Most importantly, this regards only a main effect and not a critical interaction. Did the authors not power for these? If not, that’s fine. Just label the study as an initial pilot or exploratory.

From my own calculations, the power simulations don’t look good. Assuming a correlation of 0.5 for within-subjects variables and a medium effect of d= .5 for the interaction, there is only 1.6% power to detect the interaction. This ups to 10% when we assume that the correlation between within-subjects conditions are .8.

This makes me wonder if their data are suitable for examining the interaction. That does not mean abandoning the study, but instead labelling as exploratory (like you mention in the conclusions) or a pilot and then taking this into account when encountering the range of null effects.***

Response: Thank you for raising this comment. The unit of the effect size was Cohen’s d, which was the post-hoc power. The effect size was calculated based on the difference in prosaccade latency between 5^o^ leftward and rightward targets in the RTL group (i.e. Arabic and Persian participants). 16 Arabic and Persian subjects had 85% power to detect a significant difference at 0.05 α level of one-tailed t-test. As the posthoc power analysis was computed based on a single prediction of a single t-test, hence the alpha value was set to 0.05. We agree with the reviewer that our study should be claimed as an “exploratory” study. Hence, this posthoc power analysis has been removed from the revised manuscript.

We appreciate this suggestion and have revised our title and manuscript throughout to indicate that this was a pilot study that provide a platform for further exploration.

***2. The authors explain one set of hypotheses and don’t elaborate on individual DVs. They should correct for multiple comparisons and use a new p-value threshold.***

Response: Thank you for bringing this point. Given that there were 6 DVs (Page 11, line 175), the p-value reported in the manuscript had already been adjusted for multiple comparisons. This information has been added on Page 11 (line 185).

***3. The authors state that two different trackers were used. Does the data quality differ given different rates (i.e. 500 Hz vs 60 Hz)? Could the authors plot data for the dependent variables between the two eye trackers to convince a sceptic? This could be supplemental materials.***

Response: Thank you for the reviewer’s comment. The data was collected at 2 sites (i.e. The University of Melbourne and The Hong Kong Polytechnic University), while the 2 eye trackers used were from the same manufacturer (SR Research, Scarborough, ONT, Canada), both with a sampling rate of 500 Hz. The differences between the two sites were the eye trackers’ model (Eyelink Portable Duo vs. Eyelink 1000) and the monitor (BenQ xl2540 vs. Dell U2711B, both with a refresh rate of 60 Hz). To address the reviewer’s concern about the potential difference of trackers used at different sites, we have conducted a mixed analysis of variance (ANOVA) with 2 eye trackers (Eyelink Portable Duo vs. Eyelink 1000) and group (LTR vs. RTL) as between-subject factors on prosaccade latency (Fig a.), prosaccade gain (Fig b.) and antisaccade latency (Fig c.).

Fig a.	Effect of different trackers on prosaccade latency

Average prosaccade latency with standard deviations was plotted for the LTR (i.e. Chinese participants) and RTL groups (i.e. Arabic and Persian participants) using 2 different eye trackers (Eyelink 1000 vs. Eyelink Portable Duo). Statistically, no significant main effect of trackers was found on prosaccade latency (F(1, 28)=0.64, p=0.43). The interaction between the trackers and group was not significant (F(1, 28)=0.07, p=0.80). Prosaccade latency recorded by the Eyelink 1000 and Eyelink Portable Duo was similar (178.84 ± 17.11 vs. 185.23 ± 29.05 msec).

Fig b.	Effect of different trackers on prosaccade gain

Similar results were found on prosaccade gain. No significant main effect of eye trackers (F(1, 28)=0.43, p=0.52) or interaction effect (F(1, 28)=0.09, p=0.77) was found. Prosaccade gain was similar between the Eyelink 1000 and Eyelink Portable Duo (0.99 ± 0.07 vs. 0.97 ± 0.13).

Fig c.	Effect of different trackers on antisaccade latency

Neither trackers (F(1, 28)=2.01, p=0.17) nor interaction (F(1, 28)=0.00, p=0.99) had significant effects on antisaccade latency towards correct directions. The latency was similar between the 2 eye trackers (272.93 ± 28.06 vs. 292.63 ± 48.74 msec).

These figures have been added as an Appendix in the revised manuscript.

***4. I cannot recommend enough that the authors should make the data available. Not only will this enable the analytic methods to be checked and improve transparency, but it is also difficult to reconcile with PLOS ONE’s mission of open research. The authors provide no real justification of why a ‘reasonable request’ determines access. What are the criteria for reasonable requests? Why isn’t the data fully available?***

Response: Thank you for this suggestion. The data are now available in the below platform for open access.

<https://figshare.com/articles/dataset/Effect_of_habitual_reading_direction_on_saccadic_eye_movements_A_pilot_study/19841758>

This information has been added on Page 23 (line 475).

***5. When interpreting the data, it might be worthwhile to take in reading architecture. Readers of Chinese make smaller saccades as character information is very dense. If reading influences eye movement behaviour across a range of scenarios, then this might explain increased directional errors in Chinese participants. This may essentially reflect habituation.***

Response: Thank you for reviewer’s suggestion. We have revised our discussion section accordingly to consider the effect of reading architecture of Chinese characters on antisaccade performance (refer to Page 21, line 423-426).

**Minor points:**

***1. In the abstract, the authors should make it clear that the participants were bilingual. This has major consequences for interpretation and so it is important to state clearly.***

Response: Thank you for raising this comment. All subjects were bilingual readers who read English as their second language. The statement has been added in the abstract.

***2. On page 4 (lines 30-35) this seems almost irrelevant to the paper as you do not ever look at clinical disorders. I would recommend omitting in a potential revision.***

Response: We agree with the reviewer’s suggestion that these sentences are not relevant to the main context of the manuscript. These have been removed on Page 4.

***3. Page 7 (line 118): why are the universities referenced? This is unclear. Was the data used in a prior study?***

Response: The data was collected at 2 sites (i.e. The University of Melbourne and The Hong Kong Polytechnic University) by the same experimenter (AQL). These data were not used in previous studies. The universities referenced on Page 8 (line 113) implied that all subjects were college-educated, with 20 of them being recruited and tested at The University of Melbourne while the remained subjects were recruited and tested at The Hong Kong Polytechnic University. We have revised this sentence to “32 young university students who were bilingual speakers/readers were recruited (20 were recruited and tested at The University of Melbourne and 16 were recruited and tested at The Hong Kong Polytechnic University)” for a clearer description.

***4. Page 8 (line 127): Please list the test used to assess differences in age. This should be done throughout the manuscript, and you should list test statistics throughout.***

Response: The age difference was assessed using the Student’s t-test. This information has been added on Page 8 (line 121). Accordingly, throughout the manuscript, the methods of the statistics used have been provided.

***5. Page 10 (data analysis): Could the authors more explicitly state the models used? I found it difficult to determine model structure here. You could use syntax to show this.***

Response: Thank you for bringing this comment. For prosaccade latency, prosaccade gain, correct antisaccade latency, and antisaccade error rate, 3-way ANOVA was used with group (LTR vs. RTL) as between-subject factor and stimulus direction (with- vs. against-reading direction) and magnitude (5^o^ vs. 10^o^) as within-subject factors. For self-paced saccades, subjects were asked to look back and forth between two 10^o^ targets which were presented at the left and right sides of the center. Hence, no effect of stimulus magnitude could be examined on the inter-saccadic interval and self-paced saccade gain. Instead, 2-way ANOVA was used with group as between-subject factor and stimulus direction as within-subject factor to examine any significant main effect or interaction effect. In addition, a further 2-way ANOVA was performed to examine if there was any interaction between groups and saccade type (prosaccade vs. antisaccade) on saccade latency. All these additional information about the statistical analyses have been added on Page 11 (line 181). Adjusted p-value of less than 0.05 was considered statistically significant.

***6. Consistent report digits in the results at either 2 or 3 decimals. Effect sizes should also be reported where possible.***

Response: As suggested by the reviewer, the digits reported in the result have been changed to 2 decimals throughout the manuscript for consistency. Effect size has also been reported accordingly.

***7. Could the authors please report all conditions in the figures? It would make assessing the evidence a lot easier*.**

Response: Thank you for this suggestion. The illustrations of the results have been changed from tables to figures accordingly in the revised manuscript.
