# Supplementary material for: Effect of habitual reading direction on saccadic eye movements: A pilot study
Source: PLoS One. 2023 Jun 2;18(6):e0286801. doi: 10.1371/journal.pone.0286801 (PMC10237664; doi:10.1371/journal.pone.0286801)
Supplement: Attachment — Submitted filename: Response to reviewers.docx [file pone.0286801.s003.docx]

**Response to reviewers**

PONE-D-22-23748

Dear Dr. Bharadwaj and Reviewers,

Thank you for giving us the opportunity to submit a revised draft of the manuscript entitled “Effect of habitual reading direction on saccadic eye movements: A pilot study” for publication in *PLOS ONE*. We appreciate your time and effort to provide feedback on our manuscript. The comments are valuable to improve the quality of our paper. We have made the corresponding changes in our manuscript, which are highlighted in yellow. Please also see below for the point-to-point response to the reviewers’ comments.

**Reviewer #1’s comments**

***1. there are a few possible confounds, some of which are mentioned in the "Limitations", but I think some points need to be reiterated.***

***For example, there are clear differences between the two languages utilized, beyond just the direction: the very different perceptual spans, and different orthographies. These are mentioned in different places in the text, but I think they should re-appear in Section 4.6 Limitations.***

***When mentioning the Chinese participants (L458-459): "..differences could be due to the culture or reading habit differences", you could have: "culture, reading habit differences, different perceptual spans and different orthographies."***

Response: Thank you for this suggestion. These possible confounds have been added to the revised limitation section to reiterate the differences in orthographies between the 2 languages (on Page 22, line 448).

***2. L463: "neural activities of oculomotor behaviours"***

***L474: "neural mechanisms of oculomotor behaviours"***

***-> I don't see how your study can tell us much about neural mechanisms (neural activities sounds strange), although it is insightful about oculomotor patterns/behaviours. I prefer backing off the "neural" or removing these lines.***

Response: As suggested by the reviewer, the “neural activities/mechanisms” have been removed on Page 23 (lines 455 and 466).

**Suggestions:**

***1. L145: "(also see S1 Appendix)." You could explicitly state in the text something like "there was no statistical difference between the results from the two eye trackers (see S1 Appendix)", rather than having the reference to the Appendix without any clue about what is provided there.***

Response: We agree with the reviewer’s suggestion. This sentence has been revised accordingly (refer to Page 10, line 149) in the revised manuscript.

***2. L148: I assume the tasks must have been blocked, but it would be good to state so explicitly.***

Response: Yes, the 3 tasks have been blocked. This information has been added to Page 10 (line 153).

***3. L340: "Chinese participants generated more low latency compared to non-Chinese participants (Caucasian participants") -> does 'Caucasian participants' add any useful information here? I would remove.***

Response: As suggested by the reviewer, the “Caucasian participants” has been removed on Page 18 (line 329).

**Minor / Grammar**

***1. Abstract, 3rd-to-last-sentence: "for stimulus appearing" -> "for stimuli appearing"***

Response: Thank you for pointing this out. The word “for stimulus appearing” has been revised accordingly throughout the abstract and main body.

***2. L55: "see below of section 1.3" -> see section 1.3 below***

Response: As suggested by the reviewer, line 55 has been changed to “see section 1.3 below” on Page 6 (line 56).

***3. L90: "Yan et al. took the advantage that" -> "Yan et al. took advantage of the fact that"***

Response: Thank you for this suggestion. This sentence has been revised accordingly on Page 7 (line 90).

***4. L108: "..compared with the target appeared.." -> "compared with when the target appeared"***

Response: This sentence has been revised as suggested by the reviewer on Page 8 (line 111).

***5. L156: "whose latency falls between" -> "whose latency fell between"***

Response: Thank you for pointing this out. The wording has been changed accordingly on Page 10 (line 161).

***6. L209: "The prosaccade latency for the LTR and RTL groups responded to 5o"
L235: (same error as above)
L248: (same error as above)
L267: (same error as above)***

Response: Thank you for raising this comment. Line 213 has been changed to “The prosaccade latency for the LTR and RTL groups when stimuli appeared at 5^o^ with-direction, 5^o^ against-direction, 10^o^ with-direction, and 10^o^ against-direction locations. Similar changes have been made on lines 238, and 250 accordingly.

Line 270 has been removed as suggested by Reviewer #2.

**Reviewer #2’s comments**

***1. Cheong et al., have investigated the habitual reading direction and its effect on saccadic eye movements. They are presenting their pilot study results here. While the authors give a nice introduction on saccades, they are unable to articulate what exactly is their study rationale. There is only one line that states this study result will help to better investigate the differences of eye movement control across populations! Why is this important? If you are projecting at a population level, you need to justify why that is the case, or are you asking a basic science question to explain some mechanism. So I am not clear at all with the study rationale here.***

Response: Thank you for raising this comment. The aim of the current study was to address a basic science question and fill in the research gap. Most previous studies investigated the effect of habitual reading direction on saccadic eye movements during the high-level reading process. However, saccades responding to low-cognitive-demand stimuli facilitate the understanding of the influence of long-term reading habits on reflexive and volitional saccade generations, as well as provide insights into cultural influences on the modulation of saccadic eye movements and spatial attention. This information has been added to the revised manuscript on Page 8 (line 101).

***2. I am also confused on the study hypothesis. The abstract states saccades in the direction of habitual reading direction would show longer prosaccade latency. Shouldn’t this be shorter? I suppose with reading one could argue both ways, as one begins to read from right to left, more saccades are planned leftwards, but the reverse sweep saccade needs to land back to right. Under this premise it would have been interesting to check the gain of the anti-saccades & check for this direction bias. I suppose one could then hypothesize that the antiscaccade gain in the direction of the reverse sweep saccade perhaps is more accurate.***

Response: Thank you for this constructive comment. Eviatar (1995) found that readers who habitually read from LTR took longer to respond to a target appearing on the side of their habitual reading direction (i.e. the right visual field) when they were required to ignore distractors presenting on the left. The same result was found in the RTL readers, where the response was slow when the target was presented in the left visual field (the side of the habitual reading direction). He concluded that reading direction affected the early process of visual perception and biased the reflexive attentional orientation towards the side where reading began (i.e. the non-habitual reading direction side). Furthermore, attention is needed to orient the target location prior to the execution of a saccade. The disengagement and engagement of attention facilitate the generation of saccades. Therefore, we hypothesized that prosaccades towards the side of habitual reading direction would have longer prosaccade latency, compared with the prosaccades towards the attention-biased side.

Nevertheless, antisaccade gain was not examined in the current study. An antisaccade requires the participants to suppress a reflexive saccade to the target and make a volitional saccade to the mirror location of the target, where no visible cue is presented to guide the landing position of an antisaccade. Unlike in reading, where backward saccades are made to reread previously fixated words. Therefore, error rates for glancing suddenly presented stimuli, rather than the accuracy in antisaccade gain were examined in the antisaccade task.

***3. I am not sure on the study design, and why those amplitudes were chosen. The primary question is hinging on the reading direction and influence of that on saccades. In that case, the participants chosen should have been tested for their reading pattern on a reading task to check for reading speed, regression saccades, reading comprehension, and perhaps even eye movement recordings to make sure they are standardized, especially when the sample size is small. The study design does not explain why the amplitudes of 5 and 10 degrees were chosen. Why are we looking at self-paced saccades etc.***

Response: Thank you for this comment. The primary aim of the current study was to investigate the effect of habitual reading direction on saccadic eye movements for low-cognitive-demand stimuli. Therefore, saccade directionality was examined when a dot appeared on the left or right side of fixation rather than eye movements during reading. In this study, 3 types of saccadic eye movements were examined: prosaccade, antisaccade, and self-paced saccade, as they included both reflexive and volitional eye movements. Self-paced saccades have been suggested to be an almost entirely volitional eye movement task that requires self-initiated refixations between 2 static stimuli. As reading involves a series of saccadic eye movements along lines of text toward one direction (and some regressive saccades in the opposite direction), we questioned if this reading practice also had an effect when saccades were made entirely volitionally in 2 directions in the absence of any active triggers, or if reading had a greater influence on reflexive eye movements. A dot appearing at 5^o^ and 10^o^ from fixation was chosen as the stimulus in the current study for 2 reasons. First, they were close to the 2 most frequent locations examined in the studies investigating the linkage between saccade and spatial attention (Fischer & Weber, 1993; Hoffman & Subramaniam, 1995; Roberts, Hager, & Heron, 1994). The selection of 5^o^ and 10^o^ allows a comparison of the current results with previous literature, thus providing insights into the effect of habitual reading direction on the modulation of attention and saccades. Second, 5^o^ stimuli exceeded the perceptual span of Chinese participants for acquiring useful information, while still falling into the perceptual span of the Arabic and Persian participants. The selection of the current stimuli allowed a comparison of the influence of different orthographies /perceptual spans on saccade initiation in addition to reading direction.

***4. I give a disclaimer that I am reviewing this paper for the first time. I understand it has been reviewed earlier and some comments of the earlier reviewer have been addressed in this version that I am reading. I realize more figures have been added as a result, but I feel that only figures that are useful to generate new knowledge or are surprising can be retained. Not every result needs a figure!***

Response: Thank you for this suggestion. Fig. 5 and 7 which showed the antisaccade error rates and inter-saccadic interval of self-paced saccades have been removed from the revised manuscript. The others remain unchanged to better visualize the effects graphically.

References:

Eviatar, Z. (1995). Reading direction and attention: effects on lateralized ignoring. *Brain Cogn, 29*(2), 137-150.

Fischer, B., & Weber, H. (1993). Express saccades and visual attention. *Behav Brain Sci, 16*(3), 553-567.

Hoffman, J. E., & Subramaniam, B. (1995). The role of visual attention in saccadic eye movements. *Percept Psychophys, 57*(6), 787-795.

Roberts, R. J., Hager, L. D., & Heron, C. (1994). Prefrontal cognitive processes: Working memory and inhibition in the antisaccade task. *J Exp Psychol, 123*(4), 374.
