# Supplementary material for: Effect of habitual reading direction on saccadic eye movements: A pilot study
Source: PLoS One. 2023 Jun 2;18(6):e0286801. doi: 10.1371/journal.pone.0286801 (PMC10237664; doi:10.1371/journal.pone.0286801)
Supplement: Attachment — Submitted filename: Response to editor.docx [file pone.0286801.s004.docx]

**Response to editor**

PONE-D-22-23748R1

Dear Dr. Bharadwaj,

We would like to express our gratitude for the opportunity to submit the revised draft of the manuscript entitled “Effect of habitual reading direction on saccadic eye movements: A pilot study” for publication in *PLOS ONE.* We appreciate your time and effort in providing valuable feedback on our manuscript.

Further to your feedback, we have made the corresponding changes in our manuscript, which are highlighted in yellow. Please also see below for the point-to-point response to the editor’s comments.

1. ***Please review your reference list to ensure that it is complete and correct.***

Response: Thank you for this suggestion. We have thoroughly reviewed the reference list and updated it accordingly to ensure its accuracy and completeness.

2. ***While the raw data is accessible through the link provided by the authors, could the authors also share the any software that may be written to analyze the data and generate the figures in the manuscript. This will be very useful for anyone else attempting to replicate the data or re-analyze the results.***

Response: The data analysis was performed using the graph-construction software - GraphPad Prism version 9.2.0.332 for Windows (GraphPad Software, San Diego, California USA, [www.graphpad.com](http://www.graphpad.com)), rather than a written program. Nevertheless, as suggested by the editor, we have uploaded our experimental programs to the open access platform, to facilitate the replication of the study for future researchers. The information has been added on Page 23 (line 469).

Thank you.

Allen MY Cheong
